# A novel PCOS rat model and an evaluation of its reproductive, metabolic, and behavioral phenotypes

**DOI:** 10.1002/rmb2.12416

**Published:** 2021-10-02

**Authors:** Shuhei Kamada, Yuri Yamamoto, Hidenori Aoki, Kou Tamura, Asuka Takeda, Saki Minato, Rie Masaki, Rie Yanagihara, Noriko Hayashi, Yuya Yano, Junki Imaizumi, Tomohiro Kagawa, Atsuko Yoshida, Takako Kawakita, Minoru Irahara, Takeshi Iwasa

**Affiliations:** ^1^ Department of Obstetrics and Gynecology Graduate School of Biomedical Sciences Tokushima University Tokushima Japan

**Keywords:** activity, DHT, metabolic, PCOS, reproduction

## Abstract

**Background:**

Although animal models of PCOS have been used in many studies, none of them can reproduce both the reproductive and metabolic phenotypes of PCOS. In addition, behavioral parameters have not been evaluated in PCOS animal models.

**Purpose:**

We tried to produce an improved rat model of PCOS, and the reproductive, metabolic, and behavioral phenotypes of the model rats were evaluated.

**Methods:**

Female rats were implanted with silicon tubes containing oil‐dissolved dihydrotestosterone (Oil‐DHT) as a new PCOS model. Their phenotypes were compared with those of conventional PCOS model rats (DHT), into which tubes containing crystalline DHT were implanted, and non‐DHT‐treated rats (control).

**Results:**

Both the Oil‐DHT and DHT rats showed greater body weight gain, food intake, and fat depot weight than the control rats. Furthermore, these groups showed fewer estrous stages and increased numbers of cystic follicles. The DHT rats exhibited lower ovarian and uterine weights than the control rats, whereas no such changes were observed in the Oil‐DHT rats. The Oil‐DHT and DHT rats showed less locomotor activity in the light phase than the control rats.

**Conclusions:**

Our proposed PCOS model reproduced both the reproductive and metabolic phenotypes of PCOS and may have potential for PCOS research.

## 1 INTRODUCTION

Polycystic ovary syndrome (PCOS) is a common endocrine disorder among women of reproductive age (estimated prevalence: 5–16%).^1^, ^2^, ^3^ The key symptoms of PCOS are anovulation, hyperandrogenism, and distinctive ovarian morphology; that is, polycystic ovaries (PCO), and it is commonly complicated with metabolic disturbances, such as obesity and insulin resistance.^4^, ^5^, ^6^ Although the etiology of PCOS remains unclear, it has been suggested that hyperandrogenism plays pivotal roles in the onset and progression of PCOS.^2^, ^7^, ^8^ Rodents that are subjected to chronic androgen treatment show PCOS‐like reproductive and metabolic features from early in life, and hence, have been used as rodent models of PCOS.^9^, ^10^ As PCOS is a systemic disease, these animal models are useful for evaluating the pathophysiology of PCOS in several tissues. On the other hand, no PCOS animal models can completely reproduce the reproductive and metabolic phenotypes of PCOS; that is, some animal models can reproduce the reproductive phenotype well, but poorly reproduce the metabolic phenotype, whereas the opposite is true of other animal models.^9^, ^10^

Among the various PCOS animal models, rats treated with dihydrotestosterone (DHT) until the prepubertal period have been used in basic research.^9^, ^10^, ^11^, ^12^, ^13^ Although this animal model reproduces some reproductive and metabolic phenotypes of PCOS well, such as an irregular estrous cycle and increases in body weight and fat depot weight, it poorly reproduces the associated changes in ovarian weight and/or ovarian morphology. Most women with PCOS show enlarged ovaries and PCO,^4^, ^14^ whereas DHT‐induced PCOS model rats show low ovarian weights and do not always exhibit an increased number of cystic follicles.^9^, ^10^, ^11^, ^12^, ^13^ In addition, in our preliminary examination it was confirmed that DHT‐induced PCOS model rats had low uterine weights. It is possible that these phenotypic discrepancies between human patients and animal models may hamper the evaluation of the pathophysiology of PCOS at the systemic level. Thus, improved PCOS rat models, which reproduce the hallmarks of PCOS more closely, are desired.

Another issue with PCOS animal models is that the safety of chronic hormonal manipulation has not been fully elucidated. Although it has been reported that the long‐term administration of androgens might have some adverse effects, such as liver and kidney toxicity, hepatic and renal function have not been measured in most androgen‐induced PCOS animal models. In addition, although it has been suggested that gonadal hormones affect locomotor activity and thermogenesis,^15^, ^16^, ^17^ these physiological parameters have not been measured in any PCOS animal model.

Usually, DHT pellets or silicon tubes containing crystalline DHT are used to produce DHT‐induced PCOS animal models.^9^, ^10^, ^11^, ^13^ However, we suspected that these procedures may result in DHT having supra‐physiological effects on tissues and organs, and may induce some of the phenotypic discrepancies observed between PCOS animal models and human PCOS, such as small ovaries and uteri. On the contrary, it has been well established that the implantation of a silicon tube containing oil‐dissolved estradiol can reproduce the physiological hormonal milieu in female rodents.^18^ Thus, in this study we tried to establish an improved PCOS rat model using a similar method; that is, the implantation of a silicon tube containing oil‐dissolved DHT. The reproductive and metabolic phenotypes of this new PCOS rat model were compared with those of a conventional PCOS rat model. In addition, hepatic and renal function were measured to confirm the safety of this new animal model, and locomotor activity and core body temperature were also assessed to evaluate whether changes in these physiological functions might be related to body weight adiposity in DHT‐induced PCOS animal models.

## 2 MATERIALS AND METHODS

### 2.1 Animals

Twenty‐three‐day‐old female Wistar rats were purchased from Charles River Laboratories Japan, Inc. (Kanagawa, Japan). They were housed in the animal laboratory of Tokushima University under a 12‐hour light/dark cycle (lights turned on at 0800 and turned off at 2000) and controlled temperature (24℃) conditions and were given free access to standard chow (type MF; Oriental Yeast Co. Ltd., Tokyo, Japan; 359 kcal/100 g, 12.8% of the provided calories were derived from fat, 25.6% were from protein, and 61.6% were from carbohydrates) and water. In total, 49 rats were used in this study (29 rats were used for experiment 1, and another 20 were used for experiment 2). The surgical procedures and tissue sampling were carried out under sevoflurane‐induced anesthesia, and all experiments were performed in accordance with the ethical standards of the animal care and use committee of Tokushima University (T2019‐76).

### 2.2 Effects of chronic DHT administration on reproductive and metabolic functions

Twenty‐nine rats were divided into conventional PCOS model (DHT, n=10), new PCOS model (Oil‐DHT, n=9), and non‐DHT‐treated (control, n=10) groups. In the DHT group, each rat was implanted with a silicon tube filled with crystalline DHT (As one Co., Ltd., Tokyo, Japan; inner diameter, 3 mm; outer diameter, 5 mm; length of the filled part, 5 mm). In the Oil‐DHT group, each rat was implanted with a silicon tube filled with diluted DHT; that is, DHT dissolved in a solution of 80% peanut oil and 20% ethanol (16 mg/mL) (As one Co., Ltd., Tokyo, Japan; inner diameter, 3 mm; outer diameter, 5 mm; length of the filled part, 10 mm). In the control group, each rat was implanted with an empty tube. All surgery was done on postnatal day (PND) 26, and the rats were housed individually after the surgery.

Body weight and food intake were measured every week. Food was placed in the food space of the wire mesh top, and the remaining food weight was measured. Wood chip bedding was changed at measurement, and broken food in the bedding was collected at this time point. In addition, estrous cyclicity was checked for ten days from PND48 to PND57. Specifically, a glass pipette filled with sterilized water was inserted into the vaginal orifice to a depth of 5 mm, and the vagina was flushed two or three times. Then, a small sample of the collected fluid was dropped onto a slide and dried in air. All of these slides were stained with Giemsa stain. Then, cytological examinations were performed, and the stages of the estrous cycle (proestrus, estrus, metestrus, and diestrus) were analyzed based on the relative numbers of each cell type in the vaginal smears.

At PND58, the rats were anesthetized and killed by decapitation after 16 h fasting. Then, their blood, visceral fat, subcutaneous fat, ovaries, and uteri were collected. Each sample was around 300–400 mm^3^ in size, and the histological tissue samples were fixed in 4% paraformaldehyde, embedded in paraffin, and stained with hematoxylin and eosin. Prior to fat sampling, the visceral and subcutaneous fat were peeled away from the surrounding tissue and weighed immediately. In addition, weights of uterus and bilateral ovaries were measured. Whole blood was centrifuged at 3000 rpm for 20 min at 4℃, and the serum was removed and stored at −40℃, before being used for the subsequent analyses.

The serum levels of blood urea nitrogen, creatinine, aspartate aminotransferase, alanine aminotransferase, glucose, triglycerides, total cholesterol, high‐density lipoprotein cholesterol, and low‐density lipoprotein cholesterol were measured by a commercial laboratory (Oriental Yeast Co., Ltd). The serum levels of estradiol, progesterone, and dihydrotestosterone were measured using liquid chromatography‐tandem mass spectrometry (LC‐MS/MS) by ASKA Pharmaceutical Medical Inc. Co. Ltd., which has higher sensitivity and accuracy than other measurement assays.

Ovarian samples (left side) were embedded in paraffin and then sliced into sections. Serial 4‐μm‐thick sections were stained with hematoxylin and eosin. The section with the largest area was chosen for analysis. The Zeiss Imager M2 microscope and the AxioVision (version 4.8) acquisition software (Zeiss) were used to capture the histological images. We captured one image from each rat. In each captured image, number of cystic follicle and corpora lutea were counted. Cystic follicles were defined as follows: large fluid‐filled cyst with an attenuated granulosa cell layer, dispersed theca cell layer, and an oocyte lacking connection with the granulosa cells.^12^

### 2.3 Effects of chronic DHT administration on locomotor activity and body temperature

The other 20 female rats were divided into DHT (n=9), Oil‐DHT (n=5), and control (n=6) groups. Appropriate tubes were implanted at PND27 as described above. Around PND50, pre‐calibrated radio telemetry transmitters (TA11TA‐F10; Data Sciences International, New Brighton, MN, USA) were surgically implanted into the rats. The rats were allowed to recover for around one week, and then their core body temperature and locomotor activity were measured for 48 h. The radio signals were recorded every 15 min and directly converted into body temperature and locomotor activity data using the DATAQUEST software (Data Sciences).

### 2.4 Statistical analyses

All results are expressed as mean ±standard error of the mean (SEM) values. Comparisons among the DHT, Oil‐DHT, and control groups were conducted using one‐way or two‐way repeated‐measures analysis of variance (ANOVA) followed by the Tukey‐Kramer or Dunnett *post hoc* test. *P*‐values of <0.05 were considered to be statistically significant in all statistical comparisons.

## 3 RESULTS

### 3.1 Effects of chronic DHT administration on reproductive and metabolic functions

**FIGURE 1:**
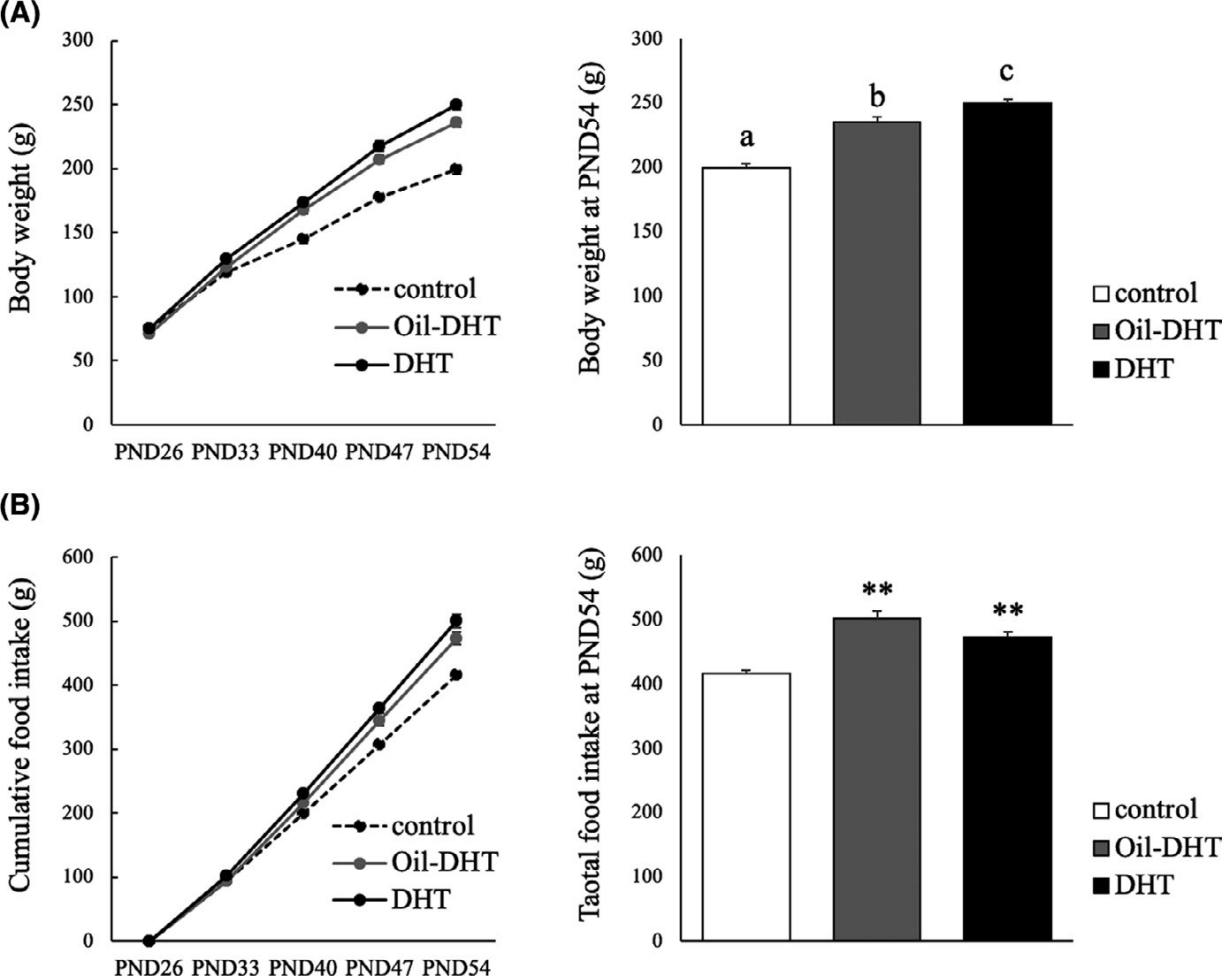
(A) Body weight change and body weight at PND54 and (B) cumulative food intake and total food intake at PND54 in the control, Oil‐DHT, and DHT groups (n=9−10 per group). Data are expressed as the mean ±SEM. ** *p* <0.01 vs. control group. Values with different letters (a−c) are significantly different (*p* <0.05)

**FIGURE 2:**
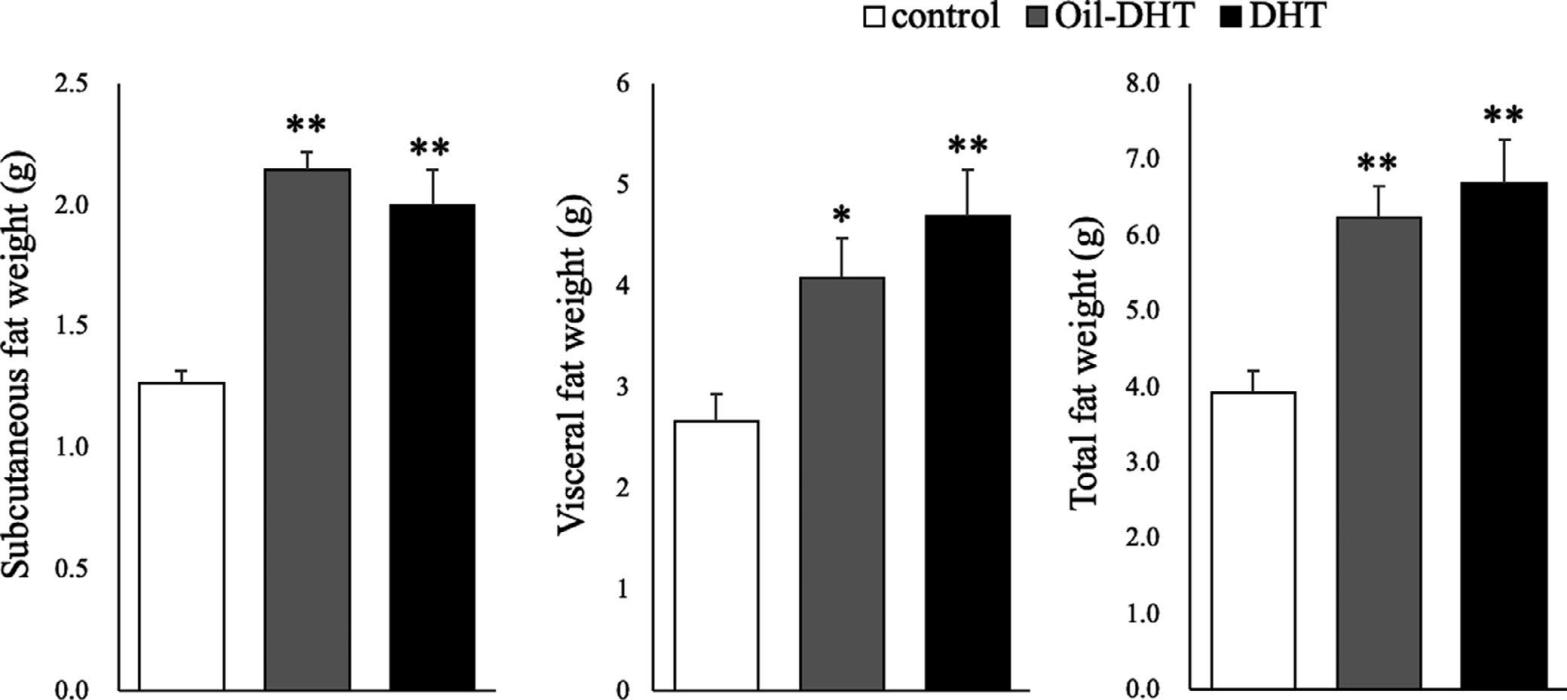
Subcutaneous, visceral, and total fat weight on the day of tissue sampling in the control, Oil‐DHT, and DHT groups (n=9−10 per group). Data are expressed as the mean ±SEM. ** *p* <0.01 vs. control group

**FIGURE 3:**
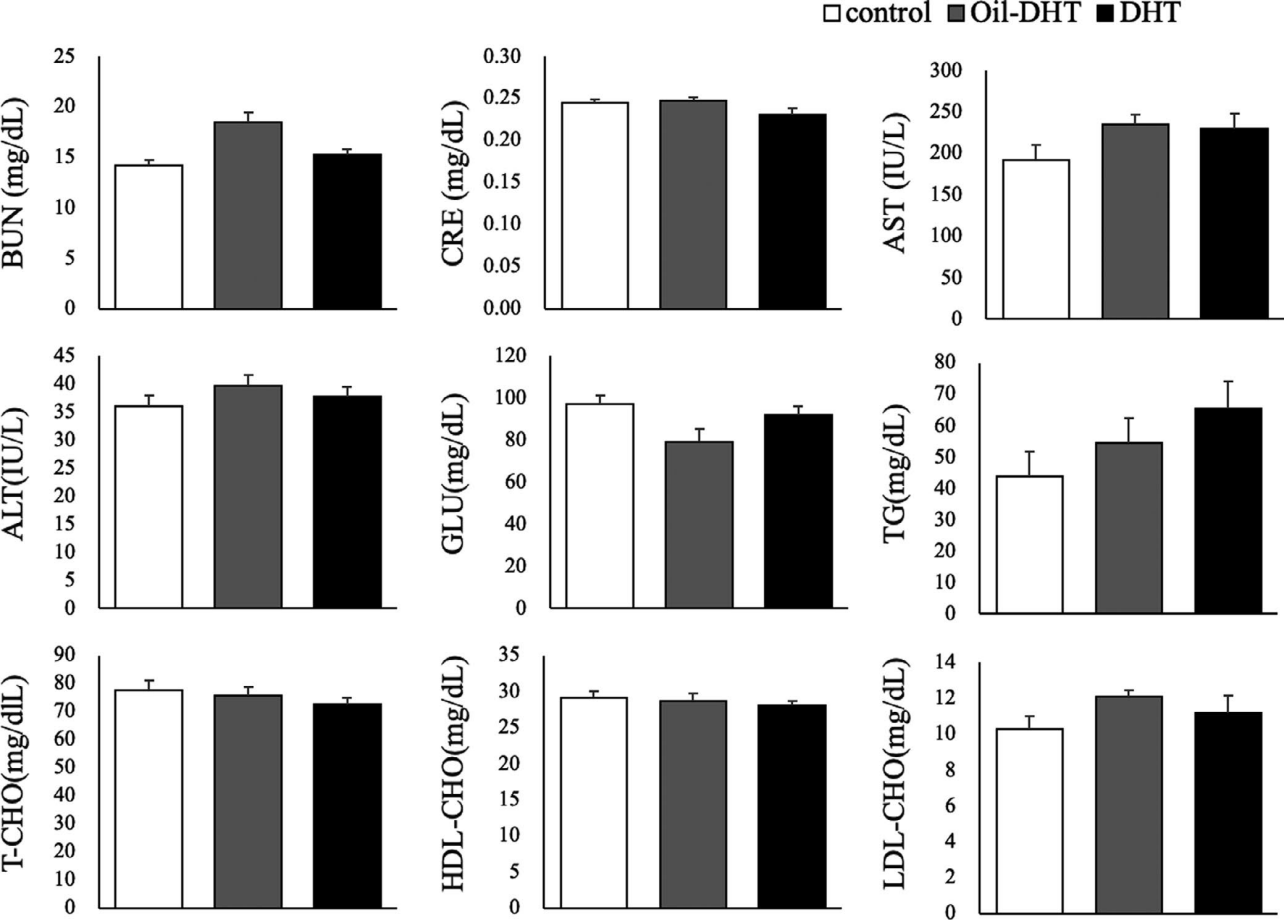
Serum levels of various biochemical factors on the day of tissue sampling in the control, Oil‐DHT, and DHT groups (n=9−10 per group). Data are expressed as the mean ± SEM

The intervention had significant effects on the observed body weight changes (two‐way ANOVA; treatment: F (2,144)=100.3, *p* <0.001; time: F (4,144)=1243.4, *p* <0.001; interaction: F (8,144)=13.8, *p* <0.001), and body weight at PND54 was heavier in the DHT and Oil‐DHT groups than in the control group (Figure 1A). In addition, at PND54 the mean body weight of the DHT group was heavier than that of the Oil‐DHT group (Figure 1A). Similarly, the intervention had significant effects on food intake (two‐way ANOVA; treatment: F (2,144)=63.9, *p* <0.001; time: F (4,144)=3870.1, *p* <0.001; interaction: F (8,144)=12.5, *p* <0.001), and the DHT and Oil‐DHT groups exhibited greater total food intake values at PND54 than the control group (Figure 1B). Subcutaneous fat weight, visceral fat weight, and total fat weight were greater in the DHT and Oil‐DHT groups than in the control group (Figure 2). The serum levels of the examined biochemical factors did not differ among the DHT, Oil‐DHT, and control groups (Figure 3).

**FIGURE 4:**
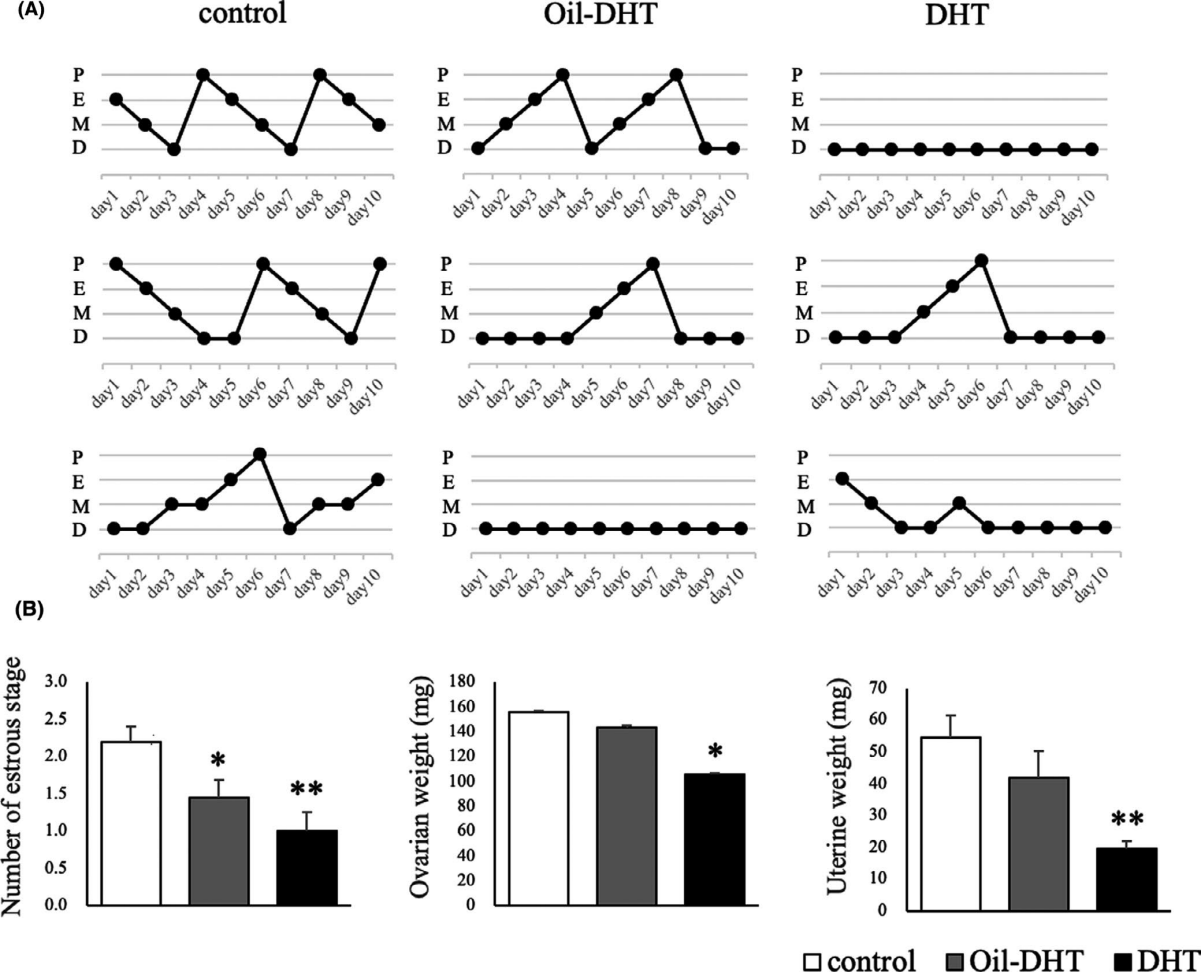
(A) The estrous cyclicity of three representative rats from each group and (B) the number of estrous stages during the 10‐day assessment period and ovarian and uterine weight on the day of tissue sampling in the control, Oil‐DHT, and DHT groups (n=9−10 per group). Data are expressed as the mean ±SEM. * *p* <0.05, ** *p* <0.01 vs. control group

Almost all of the rats in the control group showed four‐to‐five‐day regular estrous cycles, whereas many of the rats in the DHT and Oil‐DHT groups showed acyclic or irregular cycles (Figure 4A). During the 10‐day assessment period, the number of estrous stages was lower in the DHT and Oil‐DHT groups than in the control group (Figure 4B). Ovarian weight and uterine weight were lower in the DHT group than in the control group, whereas no such differences were seen between the Oil‐DHT and control groups (Figure 4B). Serum estradiol and dihydrotestosterone levels were not different among three groups, whereas serum progesterone level in Oil‐DHT and DHT groups was significantly lower than that in control group (Figure 4C).

**FIGURE 5:**
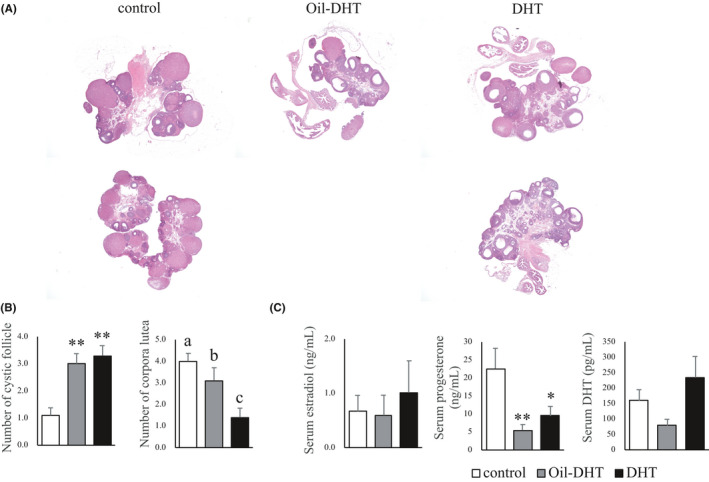
(A) The ovarian morphology of two representative rats from each group, (B) the numbers of cystic follicles and corpora lutea in ovarian tissue, and (C) serum estradiol, progesterone, and dihydrotestosterone (DHT) in the control, Oil‐DHT, and DHT groups (n=9−10 per group). Data are expressed as the mean ±SEM. * *p* <0.05, ** *p* <0.01 vs. control group. Values with different letters (a−c) are significantly different (*p* <0.05)

The ovaries of the rats in the control group had normal morphologies, whereas ovaries with a polycystic morphology were seen in the DHT and Oil‐DHT groups (Figure 5A). The number of cystic follicles was significantly larger in the DHT and Oil‐DHT groups than in the control group (Figure 5B). The number of corpora lutea was significantly lower in the DHT and Oil‐DHT groups than in the control group (Figure 5B). In addition, the number of corpora lutea was greater in the Oil‐DHT group than in the DHT group. These results are summarized in Table 1.

**TABLE 1:** Summary of the results

	Oil‐DHT	DHT
Increase of body weight gain	+	+
Increase of food intake	+	+
Increase of subcutaneous fat	+	+
Increase of visceral fat	+	+
Abnormality of biochemical tests	‐	‐
Decrease of estrous stage	+	+
Atrophy of ovary	‐	+
Atrophy of uterus	‐	+
Cystic change of ovary	+	+
Change of body temperature	‐	‐
Change of activity	+	+

### 3.2 Effects of chronic DHT administration on locomotor activity and body temperature

**FIGURE 6:**
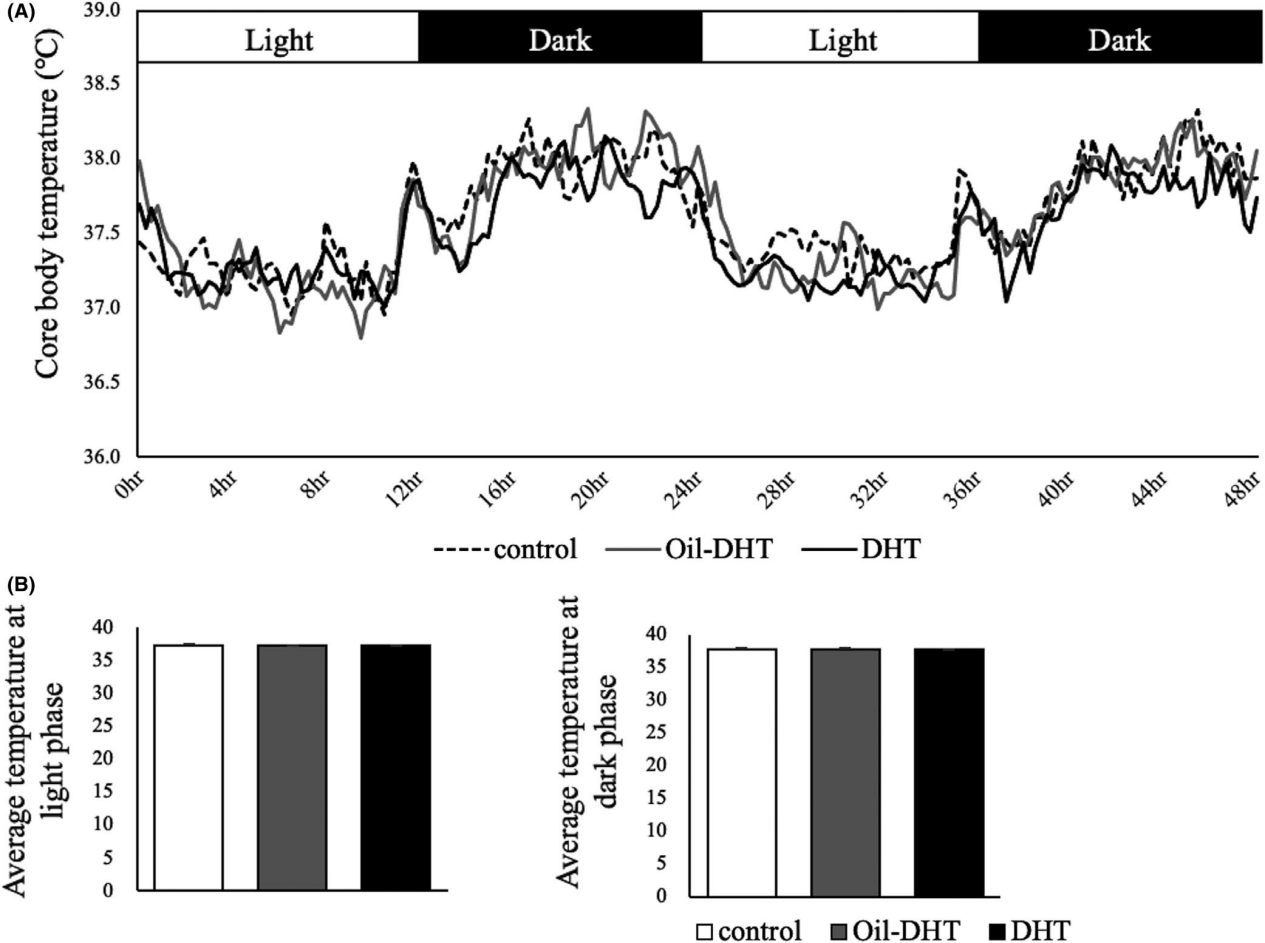
(A) Core body temperature and (B) mean temperature in the light and dark phases in the control, Oil‐DHT, and DHT groups (n=9−10 per group). Data are expressed as the mean ± SEM

**FIGURE 7:**
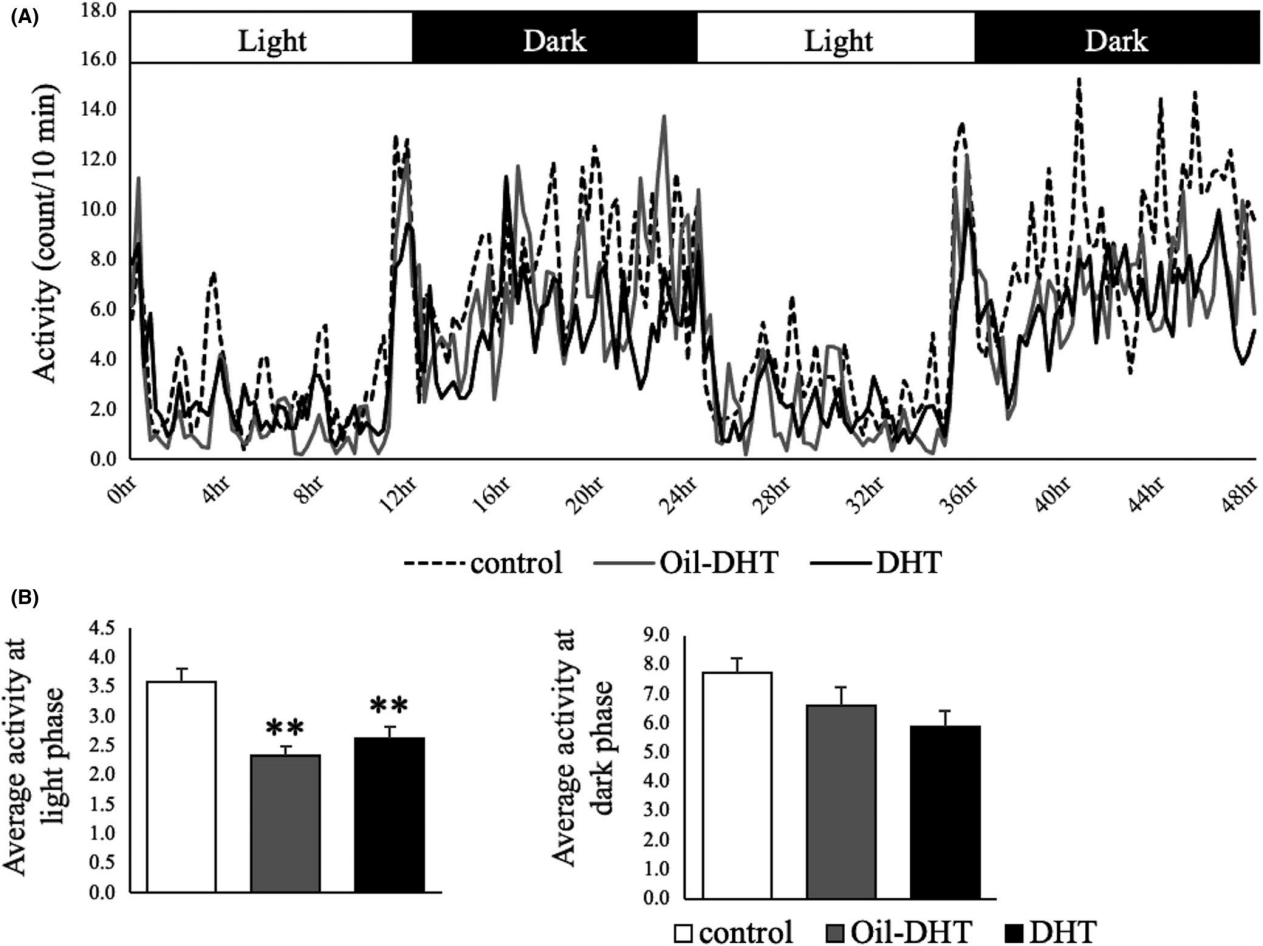
(A) Locomotor activity and (B) mean locomotor activity in the light and dark phases in the control, Oil‐DHT, and DHT groups (n=9−10 per group). Data are expressed as the mean ±SEM. ** *p* <0.01 vs. control group

Body temperature and locomotor activity exhibited circadian rhythms during the measurement period. Different patterns of change in body temperature were seen in the DHT, Oil‐DHT, and control groups (two‐way ANOVA; treatment: F (2,3989)=31.9, *p* <0.001; time: F (192,3989)=16.7, *p* <0.001; interaction: F (384,3989)=0.94, *p*=0.80) (Figure 6A). However, the mean body temperature did not differ between the light and dark phases in these groups (Figure 6B). Different patterns of change in locomotor activity were observed in the DHT, Oil‐DHT, and control groups (two‐way ANOVA; treatment: F (2,3843)=62.8, *p* <0.001; time: F (192,3843)=9.67, *p* <0.001; interaction: F (384,3843)=1.10, *p*=0.09) (Figure 7A). In the light phase, locomotor activity was lower in the DHT and Oil‐DHT groups than in the control group, whereas no intergroup differences in locomotor activity were seen in the dark phase (Figure 7B). These results are summarized in Table 1.

## 4 DISCUSSION

PCOS is a common endocrine disorder among women of reproductive age, and it is associated with reproductive disorders, such as infertility and anovulation‐related menstrual disorders, and metabolic disturbances.^1^, ^2^, ^3^, ^19^, ^20^ As noted above, various kinds of animal models have been used to evaluate the endocrine, metabolic, and histological effects of PCOS on tissues and organs.^9^, ^10^ Although DHT‐induced PCOS animal models have frequently been used for reproductive and metabolic evaluations in previous studies, they did not completely reproduce all of the phenotypes of human PCOS. For example, the DHT‐induced PCOS animal models used by Manneras et al. and our group exhibited PCO, but they also had low ovarian and/or uterine weights.^11^, ^13^, ^21^ Similarly, the DHT‐induced PCOS animal model used by Osuka et al. had small ovaries without cystic morphological ovarian changes.^12^ On the other hand, these animal models displayed disrupted ovulation and body weight and body composition abnormalities; that is, they showed disturbed estrous cyclicity and increases in body weight and fat depot weight.^11^, ^12^, ^13^, ^21^ Thus, a PCOS animal model that reproduces most PCOS phenotypes and overcomes the discrepancies between conventional animal models and human PCOS is desired.^22^

In this study, we proposed a new PCOS rat model that reproduces the reproductive and metabolic features of PCOS without any atrophic changes in the ovaries or uterus. In addition, similar to those used in previous studies, this model reproduces most PCOS phenotypes; that is, increases in body weight gain, food intake, and fat depot weight; disturbed estrous cyclicity; and PCO morphology.^9^, ^10^ In our previous studies, PCOS model rats were created by implanting a silicon tube containing crystalline DHT into each rat, whereas a tube containing dissolved DHT was implanted into each rat in this study.^11^, ^13^ Although the reasons why ovarian and uterine weight are decreased in conventional DHT‐induced PCOS model animals remain unclear, we speculate that DHT may have supra‐physiological effects on these organs in PCOS animal models. If this was true, it is possible that the pathophysiological mechanisms underlying other metabolic and reproductive features in PCOS animal models might differ from those seen in human PCOS. To confirm this hypothesis, we measured serum DHT concentrations in this study; however, we did not detect any differences in serum DHT levels among the three groups by both non‐RIA (data not shown) and LC‐MS/MS methods. As the chemical structure of the DHT used in this study (stanolone) may be slightly different from that of natural DHT, the antibody used in the non‐RIA measurement system and LC‐MSMS methods may not be able to recognize implanted DHT. Further studies are needed to clarify the mechanisms underlying and experimental utility of our new PCOS rat model.

Other problems with PCOS animal models include the fact that the safety of chronic hormone manipulation has not been fully evaluated and that changes in locomotor activity and thermogenesis have never been examined in such models. It is well known that the long‐term administration of androgens often has adverse effects and that gonadal hormones, for example, estrogen and progesterone, affect thermogenesis in females, including experimental animals.^16^, ^17^, ^23^ In addition, it has been reported that estrogen increases locomotor activity in female rodents. Female rats show the greatest locomotor activity in the proestrus stage, when they also display concomitant increases in serum estrogen levels, and estrogen supplementation increases locomotor activity in ovariectomized female rats.^24^, ^25^, ^26^ These effects of estrogen on locomotor activity are not observed in mice lacking the estrogen receptor.^18^ On the other hand, the effects of androgens on female locomotor activity, especially in androgen‐induced PCOS model animals, have not been evaluated. If the core body temperature and/or locomotor activity of PCOS model animals are altered, such changes may be related to the metabolic disturbances seen in such animals.

Thus, in this study we evaluated the safety of chronic DHT administration, as well as locomotor activity and body temperature, in new and conventional DHT‐induced PCOS model rats. As a result, we found that hepatic and renal function and core body temperature were not affected by the administration of DHT in these animal models. On the other hand, locomotor activity was decreased in the light phase, but not in the dark phase, in both the new and conventional models. In rodents, food intake and locomotor activity are both increased in the dark phase, whereas they are decreased in the light phase, during which most time is spent sleeping.^27^ It is possible that in the present study, both types of PCOS model rats spent more time sleeping or exhibited decreased activity, even when awake, and that these changes might be, at least in part, related to the increases in body weight gain and fat depot weight they exhibited. In addition to metabolic, endocrine, and immunological evaluations, examinations focusing on behavioral phenotypes are needed to clarify the pathophysiology of the metabolic disturbances seen in PCOS.

In conclusion, we proposed a new DHT‐induced rat model of PCOS, which reproduces both the reproductive and metabolic phenotypes of PCOS well. In particular, this model does not induce atrophic changes in the ovaries or uterus, which are observed in conventional DHT‐induced PCOS model animals. We also clarified that locomotor activity was decreased in both the new and conventional PCOS model rats, indicating that some behavioral changes might be related to the metabolic disturbances induced by PCOS.

## CONFLICTS OF INTEREST

The authors declare that no conflicts of interest exist.

## ETHICAL APPROVAL

This article does not describe any experiments involving human participants. All of the institutional and national guidelines for the care and use of laboratory animals were followed. The protocol for the research project was approved by a suitably constituted ethics committee.
